# Spatial Wavefunction Characterization of Femtosecond Pulses at Single-Photon Level

**DOI:** 10.34133/2020/2421017

**Published:** 2020-06-15

**Authors:** Billy Lam, Mohamed ElKabbash, Jihua Zhang, Chunlei Guo

**Affiliations:** The Institute of Optics, University of Rochester, Rochester, New York 14627, USA

## Abstract

Reading quantum information of single photons is commonly realized by quantum tomography or the direct (weak) measurement approach. However, these methods are time-consuming and face enormous challenges in characterizing single photons from an ultrafast light source due to the stringent temporal mode matching requirements. Here, we retrieve the spatial wavefunction of indistinguishable single photons from both a continuous wave source and a femtosecond light source using a self-referencing interferometer. Our method only requires nine ensemble-averaged measurements. This technique simplifies the measurement procedure of single-photon wavefunction and automatically mode matches each self-interfering single photon temporally, which enables the measurement of the spatial wavefunction of single photons from an ultrafast light source.

## 1. Introduction

In quantum optics, spatially reshaping various degrees of freedom of a single photon, e.g., amplitude, wavefront, polarization, and orbital angular momentum, has been routinely performed using classical techniques, such as holography, spatial light modulators, polarization optics, and q-plates [1–7]. Accurate tailoring of single photons enables them to function as information carriers for applications in quantum key distribution, quantum entanglement, and quantum computation [1–11]. These applications are of great importance for quantum information technologies as quantum key distribution secures the sharing of secret quantum information against eavesdropping [3–6]; quantum entanglement enables measurements beyond the standard quantum limit, i.e., shot-noise limit [10, 11]. Quantum computers solve certain problems at greater speeds than are possible with classical computers [8, 9]. All these applications require reading quantum information of single photons [1, 2, 7, 12]. Temporally localized optical pulses from ultrafast light sources are a desirable choice for many of these applications because they have a higher data rate due to the high repetition rate in time and broadband in frequency, enabling wavelength division multiplexing [13–15]. Consequently, the characterization of single photons from ultrafast light sources is of major technological importance.

Measuring the complex-valued wavefunction Ψ requires multiple measurements of an ensemble of identically prepared quanta because of the uncertainty principle, and the measurement outcome of any observable must be a real value. Currently, Ψ is usually measured by quantum tomography [16–18] in phase-space representation as a Wigner function or the direct (weak) measurement approach [19–22]. Unlike spatial wavefunction, the Wigner function of a transverse spatial quantum state is a function of both space and spatial frequency [17]. As a result, the Wigner function offers a very distinct viewpoint on wavefront aberrations. While the effects of low-order aberrations on the Wigner function have been explored, close-form analysis of higher-order aberrations is very difficult and numerical simulations are needed [23]. Optical homodyne tomography can also reveal the photon number statistic of the source by measuring marginal distributions and reconstructing the Wigner function expressed in terms of the position and momentum of a harmonic oscillator [16, 18]. Although quantum tomography can reveal additional information of quantum statistics, quantum tomography is not efficient and extremely time-consuming for extracting spatial wavefunction when the dimension *d* of the state is large. Quantum tomography involves a diverse collection of ensemble-averaged measurements proportional to *d*^2^ − 1 and computationally complex postprocessing with a vast amount of fitting parameters, becoming prohibitively difficult as *d* grows [20]. On the other hand, the direct measurement approach is a simpler alternative [19–22, 24]. However, it still requires a large number of ensemble-averaged measurements proportional to *d* [19–22], although attempts have been made to further reduce the amount of required measurements using compressive sensing [20] or array detectors [24]. Recently, a single-photon holography technique [25] has been demonstrated to retrieve an unknown wavefunction of a single photon using a known reference photon, but only along a single axis. Furthermore, it is challenging to apply the aforementioned techniques to ultrashort pulses. For example, the insertion of a tiny waveplate for weak measurement can introduce extra optical path length greater than the coherence length of the ultrashort pulses, leading to loss of interference. Ultrashort pulse interference requires matched dispersion, matched spectrum, and near-zero optical path difference. These stringent temporal mode matching conditions are difficult to satisfy.

Here, we present a common-path dispersion-matched shearing interferometer (CDSI) to measure the spatial wavefunction of single photons. To overcome the challenges associated with low photon counts, we develop a novel wavefront extraction technique without curve fitting and a phase retrieval technique with an uncertainty that scales as *N*^(−1/2)^, where *N* is the number of detected photons. Only nine ensemble-averaged measurements are required for an arbitrary *d*, greatly simplifying the measurement of the spatial wavefunction of single photons from a continuous wave laser and a 56 nm broadband ultrafast light source. Each self-interfering single photon from the ultrafast light source is temporally mode matched automatically with high fringe visibility, a condition rarely satisfied by shearing interferometers [26]. Moreover, the self-referencing property of the device circumvents the indeterminate absolute phase problem [18, 27]. In addition, our interferometer is insensitive to vibration, providing long-term stability over an indefinite time span for single-photon experiments.

**Figure 1:**
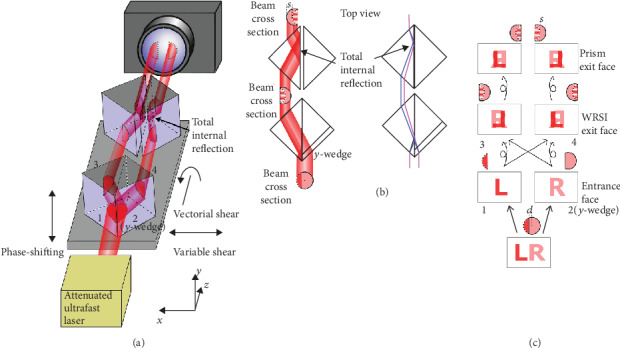
The purposed common-path dispersion-matched shearing interferometer for spatial wavefunction characterization of single photons. (a) Schematic diagram of the experimental setup. The *y*-wedge angle is highly exaggerated. Multiple neutral density filters are used to attenuate the laser beam. (b) Top view of the ray diagram showing how the right angle prisms bring the interferogram closer to the optical axis and cancel the spatial chirp induced by the CDSI. Two rays with distinct colors (red and blue) representing different wavelengths are shown. (c) Wavefront transformation after propagating through the BSC surfaces. Curly (straight) arrow indicates reflection (transmission).

Figure 1(a) presents a schematic of the experimental setup. A Ti:Sapphire mode-locked oscillator is used to generate a collimated beam as a continuous wave or ultrashort pulses with a central wavelength of 800 nm, a pulse duration of 106 fs, and a bandwidth of 56 nm at an 89 MHz repetition rate. The beam passes through a series of neutral density filters, attenuating to a single-photon level with a photon rate of 0.3 photons per meter for continuous wave operation or ∼1 photon per pulse for pulsed operation. The beam propagates through the CDSI, where the left and right parts of the beam interfere by flipping one part of the beam onto the other (Figure 1(b)). The lateral shear is controlled by translating the beam splitter cube along the shearing direction. The detailed operation principle of the CDSI can be found in Materials and Methods. The output interferograms from the CDSI are imaged onto an optically gated intensified charge-coupled device (ICCD) camera (model iStar DH734). The gated pulse that activates the intensifier tube of the ICCD camera is set to the oscillator's pulse-to-pulse separation time, so each camera frame captures exactly one pulse during pulse operation. The probability of two photons landing on the same pixel is negligible (≪10^−3^). For our particular beam intensity and camera settings used, the probability of two photons landing on the same pixel in a frame is <0.00065 (<0.00056) for continuous wave (pulsed) operation. The derivation of these probabilities is detailed in Materials and Methods.

It is very important for the recorded interferogram to be free of spatial chirp induced by the beam splitter cube (BSC). To cancel out the spatial chirp, two right angle prisms of the same size as the CDSI are placed after the CDSI to induce the opposite spatial chirp. This is depicted in Figure 1(b). Only the rays through one of the exit faces are shown. The right angle prisms are separated by a small air gap causing total internal reflection. The two right angle prisms after the CDSI also bring the two pairs of interferograms significantly closer to each other, leading to a smaller subregion on the camera and a faster camera frame rate. This setup shows similarity to the wedged reversal shearing interferometer in Ref. [28]. However, the spatial chirp issue discussed above was not resolved in Ref. [28]. Furthermore, the previous technique cannot be applied to single photons due to the stringent requirements for single-photon measurement. An interferogram that accumulates many single photons can have significant variations in counts at adjacent pixels, causing an inaccurate curve fit that ruins the wavefront extraction technique in the previous work.

In our setup, an ensemble of photons with identical initial wavefunction Ψ_*i*_(*x*, *y*) is bisected by the hypotenuse face of a single 50 : 50 beam splitter cube (BSC) with a wedged entrance face. The wavefunction propagates through both entrances, face 1 and face 2, of the BSC. The wavefunction in each side of the BSC refracts and then irradiates the hypotenuse surface, then splits and recombines simultaneously, resulting in two interfering output beams from each exit face, i.e., face 3 and face 4 (see Figure 1(c)). The final wavefunction at exit face 3 (4) is the superposition of the left reflected (transmitted) portion and the right transmitted (reflected) portion of the initial wavefunction. The probability distribution measured after exit face 3 is the square modulus given by

(1) $$\begin{array}{c} {\left|\Psi_{3}\left(x,y\right)\right|}^{2}=I^{\prime }\left(x,y\right)+I^{\prime \prime }\left(x,y\right)\cos\left(\phi \left(x,y\right)\right),\;x\geq d, \end{array}$$

where *I*′(*x*, *y*) = (|Ψ_*i*_(−*x* − *s*, *y*)|^2^ + |Ψ_*i*_(*x*, *y*)|^2^)/2 is the average of the probability density of the two portions of the incident wavefunction, *I*^″^(*x*, *y*) = |Ψ_*i*_(−*x* − *s*, *y*)||Ψ_*i*_(*x*, *y*)| is the geometric mean of the probability density of the two portions of the incident wavefunction, and *ϕ*(*x*, *y*) is the phase difference between the interfering beam. In the final expression, we do not propagate the initial wavefunctions because the entrance plane of the BSC is imaged. We assume perfect temporal coherence even for pulse duration in the femtosecond regime because CDSI guarantees near-zero optical path difference and group velocity dispersion due to near-equal arm length in the same medium [28]. Hence, the self-interfering left and right portions of the wavefunction are automatically mode matched temporally. The visibility is measured to be *V* = 0.88 ± 0.08 for continuous wave operation and *V* = 0.87 ± 0.08 for pulsed operation. The visibility is similar because the dispersion is matched very well in our device, and the beam uniformity is degraded in continuous wave operation due to cavity optimization for mode locking. Meanwhile, spontaneous parametric downconversion- (SPDC-) based heralded single-photon sources typically require spectral filtering for high indistinguishability and visibility, which excludes ultrafast (broadband) light sources [29]. For example, a SPDC source pumped by femtosecond pulses without any bandpass filter and a BBO (*β*-BaB_2_O_4_) nonlinear crystal with a typical thickness of 1 mm has a visibility of ∼0.5 [29]. The visibility can be increased to 0.7 and near 1 using a 6 nm and 0.6 nm bandpass filter, respectively [29]. The bandpass filter, however, significantly broadens the pulse duration as it is inversely proportional to bandwidth.

The probability distribution |Ψ_3_(*x*, *y*)|^2^ reveals the information about the wavefront of the unknown photon *W*(*x*, *y*) because the phase consists of the directional derivative of the wavefront as follows:

(2) $$\begin{array}{c} \phi \left(x,y\right)=\left(n-n_{\text{air}}\right)t\left(y\right)+s\frac{\partial W_{\text{o}}}{\partial x}-s\frac{\partial W_{\text{e}}}{\partial x}+2W_{\text{o}}\left(x,y\right), \end{array}$$

where *t*(*y*) = 2*πy*sin*α*/*λ* + *t*(0) ≈ 2*πyα*/*λ* + *t*(0) is the thickness difference measured in radians of the two entrance faces resulting from the *y*-wedge angle *α* of face 2 (see Figure 1) and the function *W*(*x*, *y*) is the wavefront of the incident beam, which is separated into odd and even order terms *W*_o_(*x*, *y*) and *W*_e_(*x*, *y*). The full derivation is described in Materials and Methods. By measuring two orthogonal directional derivatives using four interferograms with the shearing amounts of *s*_*x*_ = 0, *s*_*x*_ = *s*_*x*_, *s*_*y*_ = 0, and *s*_*y*_ = *s*_*y*_, where the subscripts denote the shearing direction, the wavefront can be retrieved with uncertainty up to an unknown absolute phase. Retrieving the wavefront using CDSI involves an integration of the derivatives in Equation (2) that always results in an unknown constant term (see Materials and Methods). This unknown constant term is set to zero (*W*(0, 0) = 0). As a result, *W*(*x*, *y*) is exactly the wavefront shape in the absence of the random absolute phase and can be found even for Fock states, where the photon number is known [18].

After circumventing the random absolute phase issue, we will now discuss the phase retrieval and wavefront extraction. All interferometric phase retrieval techniques require a minimum of three measurements of intensities, as there are three unknowns in the two-beam interference Equation (1). Accordingly, the phase extraction is an algebraic expression that consists of at least three intensity distributions. For example, the 4-bin phase shifting [26] phase retrieval depends on the intensity distribution at four phase-shift values; thus, the calculated phase suffers from photon number fluctuations between the four intensity distributions.

To enhance the accuracy of the measurement, we eliminate the uncertainty due to photon number fluctuation by normalizing each interferogram over the total photon number, which is measured by detecting both outputs of the BSC. This relies on the fact that the total probability over the exit faces 3 and 4 must add up to one due to energy conservation. After normalization, each intensity distribution becomes a probability distribution and the normalized 4-bin phase retrieval [26] becomes

(3) $$\begin{array}{c} \phi \left(x,y\right)=\tan^{-1}\left(\frac{\mathrm{Pr}\left(x,y;\left(3\pi /2\right)\right)-\mathrm{Pr}\left(x,y;\left(\pi /2\right)\right)}{\mathrm{Pr}\left(x,y;0\right)-\mathrm{Pr}\left(x,y;\pi \right)}\right), \end{array}$$

where Pr(*x*, *y*; *δ*) denotes the probability distribution of the interferogram with phase shift *δ* (see derivation in Materials and Methods). We note that, in general, this type of error reduction by normalization can be applied to most interferometric phase retrieval methods. In practice, certain interferometers (i.e., Michelson, Sagnac, and diffraction type) require some minor modification in order to apply the error reduction (see Materials and Methods).

We utilize the normalized 4-bin phase retrieval method (Equation (3)) to measure the phase distribution and then extract the actual wavefront by manipulating the phase. The BSC can be translated along the wedge direction to induce the phase shift required in the phase retrieval method (see Video 1). We test this retrieval method by measuring the wavefront of a focusing ultrafast laser beam at a single-photon level and the phase modulation of a one-dimensional transmissive spatial light modulator (SLM). The focusing single-photon beam is obtained by passing a collimated beam through a lens with a focal length of 150 mm. The CDSI outputs two interferograms displaced from one another, which are brought significantly closer together to be captured by the ICCD camera by passing through another right angle prism.

## 2. Results

**Figure 2:**
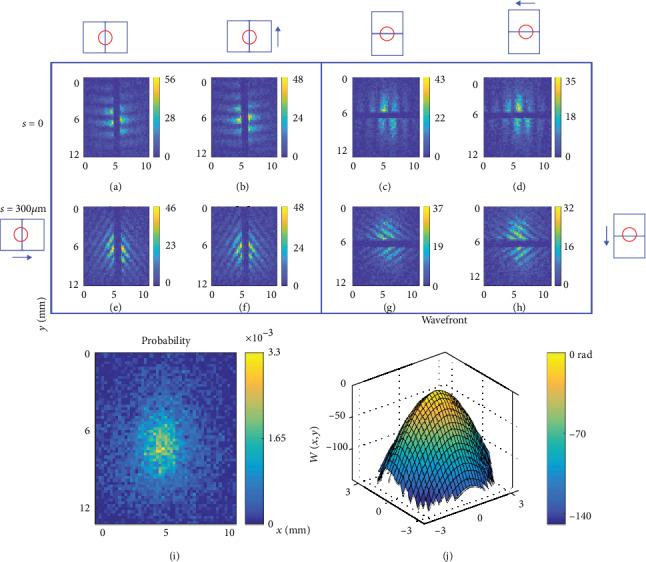
Spatial wavefunction characterization of single photons from an ultrafast light source with quadratic wavefront shape. The 4-bin phase shifting induced by translation of BSC (see Video 1) is applied to the interferograms with shearing amount of *s*_*x*_ = 0, *s*_*x*_ = 300*µ*m, *s*_*y*_ = 0, and *s*_*y*_ = 300*µ*m to retrieve the phases. The phases are manipulated numerically to extract the wavefront shape (see Materials and Methods for details of the data acquisition and wavefront extraction). (a–h) Interferograms produced by a focusing beam with a focal length of *f* = 150 mm that passed through the CDSI. The shearing amount are (a–d) *s* = 0 and (e–h) *s* = 300*µ*m. The shearing direction are (a, b, e, f) $\overset{\wedge }{x}$ and (c, d, g, h) $\overset{\wedge }{y}$. The phase shift pairs are (a, c, e, g) 0 and *π* and (b, d, f, h) *π*/2 and 3*π*/2. (i) Probability distribution of the single photon measured by translating the BSC to fully contain the beam. (j) Extracted wavefront based on the interferograms (a–h). Each interferogram is made up of accumulation of 15000 frames at the rate of 0.72 photons per frame. (See Video 2 for the accumulation of frames.)

Figure 2 demonstrates the probability density measurement as well as the wavefront retrieval of the focusing single photons from a femtosecond laser. The pairs of interferograms from the two outputs of the CDSI are out of phase of each other, so the phase-shift pairs of 0 and *π* (*π*/2 and 3*π*/2) are produced by default (translation of the BSC). Interferograms with shearing amounts of *s*_*x*_ = 0, *s*_*x*_ = 300*µ*m, *s*_*y*_ = 0, and *s*_*y*_ = 300*µ*m are taken to extract the wavefront by manipulating the phase. The details of the calculation are derived in Materials and Methods. While the interferograms (Figures 2(a)–2(h)) provide the wavefront information (Figure 2(j)), the BSC can be translated to fully contain the beam at one entrance face for measuring the probability distribution (Figure 2(i)). It is important to note that the full characterization of Ψ(*x*, *y*)  using the CDSI technique requires nine ensemble-averaged measurements. Initially, the amplitude information is obtained by imaging the incident single-photon beam through one face of the CDSI. Subsequently, the phase information is obtained by intercepting the beam using the CDSI and capturing the images of the interferograms, which requires eight ensemble-averaged measurements. In principle, the wavefront can also be extracted by curve fitting using only the interferograms with shearing amounts of *s*_*x*_ = 300*µ*m and *s*_*y*_ = 300*µ*m [28]. However, this can result in severe fitting errors for single photons.

**Figure 3:**
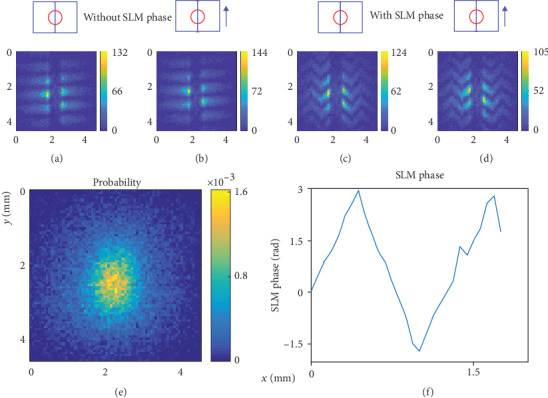
Spatial wavefunction characterization of single photons with a triangular wavefront shape. The 4-bin phase shifting induced by translation of BSC (see Video 1) is applied to the interferograms with shearing amount of *s*_*x*_ = 0 to retrieve the phases. The phases are manipulated numerically to extract the 1-dimensional odd order SLM phase distribution (see Materials and Methods for details of the wavefront extraction). (a–d) Interferograms produced by a collimated beam going through the CDSI at *s*_*x*_ = 0 (a, b) without SLM phase modulation and (c, d) with SLM triangular phase modulation. The phase shift pairs are (a, c) 0 and *π* and (b, d) *π*/2 and 3*π*/2. (e) Probability distribution of the single photon measured by translating the BSC to fully contain the beam. (j) Extracted SLM phase based on the interferograms (a–d). (See Materials and Methods for details of the wavefront extraction.) Each interferogram is made up of accumulation of 60000 frames at the rate of 0.72 photons per frame.

We have also imprinted a triangular phase distribution onto the right half of a beam using a one-dimensional SLM. The phase modulation from the SLM is retrieved by the CDSI (see Figure 3). We measure the interferograms of the beam with and without the SLM phase modulation and then take the difference to extract the SLM phase modulation. This removes the wavefront from the beam itself, leaving only the SLM phase modulation.

Only the interferograms at shearing amount of *s* = 0 are used to extract the phase because it contains the full information of odd order wavefront, sufficient to derive the SLM phase modulation that is only on the right half of the beam. The details of the calculation of the odd order wavefront are derived in Materials and Methods. The same data is also taken in continuous wave operation of the oscillator. The results are nearly identical with a slightly different beam profile (see Fig. S8). The same experiment is also performed using a sinusoidal phase distribution, and the results are reported in Figs. S6 and S7. In addition to precision wavefront measurement, CDSI can be used as an alignment tool as asymmetric wavefront aberrations show up on the interferogram at a shearing amount of *s* = 0. Meanwhile, the symmetric wavefront aberrations cause the interferogram to evolve as the shearing amount is changed. The CDSI, along with right angle prisms, can be inserted into any beam path prior to a camera to directly diagnose aberration. Such configuration introduces no beam deflection or displacement, which is great for aligning and diagnosing single-photon beams. Typical interferograms due to misalignment of lens are shown in Fig. S10.

## 3. Discussion

Our accuracy enhancement technique of normalization yields an uncertainty that scales with *N*^(−1/2)^. Normalization turns intensity distribution into probability distribution, where the probability of each pixel can be modeled by a normalized binomial distribution. Having known the photon number *N* detected in the experiment, the error in the measured value of probability *p*_*i*,*j*_ at each pixel located at (*x*_*i*_, *y*_*j*_) is $\sqrt{p_{i,j}\left(1-p_{i,j}\right)/N}$, which is derived from the standard deviation of the binomial distribution $\sqrt{Np\left(1-p\right)}$. It can be shown that after normalization, the uncertainty scales as the standard quantum limit of $1/\sqrt{N}$ for any type of phase retrieval that involves multiple measurements of intensity (probability) distribution. Let Pr_*k*_(*x*, *y*) be the probability distribution of the *k*^th^ intensity measurement. The measured phase can be written as some function of the intensity measurements:

(4) $$\begin{array}{c} \phi \left(x,y\right)=f\left({\mathrm{Pr}}_{1}\left(x,y\right),{\mathrm{Pr}}_{2}\left(x,y\right),\cdots ,{\mathrm{Pr}}_{k_{\max}}\left(x,y\right)\right). \end{array}$$

We can apply the variance formula of error propagation to find the phase error as follows [30]:

(5) $$\begin{array}{c} \Delta \phi \left(x,y\right)=\sqrt{\overset{k_{\max}}{\underset{k=1}{\sum }}{\left(\frac{d\phi \left(x,y\right)}{d{\mathrm{Pr}}_{k}\left(x,y\right)}\right)}^{2}{\left[\Delta {\mathrm{Pr}}_{k}\left(x,y\right)\right]}^{2}}. \end{array}$$

The absolute error of ΔPr_*k*_(*x*, *y*) is $\sqrt{{\mathrm{Pr}}_{k}\left(x,y\right)\left(1-{\mathrm{Pr}}_{k}\left(x,y\right)\right)/N_{k}}$ where *N*_*k*_ is the total number of photons over all outputs detected for the measurement of Pr_*k*_(*x*, *y*). The derivative term is independent of *N*_*k*_ because both *ϕ*(*x*, *y*) and the probability distribution Pr_*k*_(*x*, *y*) are independent of *N*_*k*_. Given that the total number of photons detected of each intensity measurement is the same, the phase noise Δ*ϕ*(*x*, *y*) scales as $1/\sqrt{N_{k}}$, the standard quantum noise limit. The typical value of *N*_*k*_ in our experiment is 10800 or 43200 as each measurement set of an interferogram is either an accumulation of 15000 frames or 60000 frames with an average of 0.72 photons per frame for pulsed operation.

Under this detection scheme and accuracy enhancement, an attenuated beam [1, 24, 31, 32] is a single-photon source as good as the SPDC heralded source. This can also be understood with a very simple analog: coin tosses can always be considered as binomial distribution even if the total number of coin tosses in the experiment fluctuates. The error only depends on the total number of coins tossed, but not the fluctuation of it.

We have conceptualized and demonstrated the use of the CDSI to retrieve the amplitude and wavefront of a single photon, free from complicated sets of measurements and computations such as maximum likelihood estimation and least-squares fitting. This is possible because single-photon interference, diffraction, and propagation follow classical theory of light [33, 34]. The self-referencing device guarantees automatic temporal mode matching, making it more user-friendly and applicable to various light beams including ultrafast light beams at a single-photon level. No prior information about the shape of the single-photon wavefunction is needed. Furthermore, the interferometer is very simple and easy to align as it consists of only a single beam-splitter cube. The device itself can be used as a portable visual diagnostic tool to probe wavefront aberrations of spatial wavefunctions even under the single-photon regime, making the alignment of any single-photon experiments significantly easier. Moreover, the accuracy is enhanced to scale as *N*^−(1/2)^. This accuracy enhancement technique is universal, and it can be applied to most existing interferometric phase retrieval techniques. The ability to fully characterize the spatial structure of the wavefunction will enable future research in tackling the complexity of a single photon in the spatial domain, benefiting the fields of free-space quantum communication, quantum information processing, and more.

## Data Availability

All data needed to evaluate the conclusions in the paper are present in the paper. Raw data and MATLAB codes for data processing may be requested from the authors.

## Supplementary Materials

Supplementary 1 Table S1: measured probability *p* of all possible outcomes weighted on the probability of occurrence for photon number distribution. Fig. S4: phase shifting methods of the CDSI. Fig. S5: fringe pattern of the CDSI for a collimated beam recorded by the CCD camera when the image plane of the CCD lens and the entrance plane of the BSC are not conjugate of each other. Fig. S6: spatial wavefunction characterization of femtosecond-pulsed single photons with a sinusoidal wavefront shape. Fig. S7: spatial wavefunction characterization of CW single photons with a sinusoidal wavefront shape. Fig. S8: spatial wavefunction characterization of CW single photons with a triangular wavefront shape. Fig. S9: wavefront retrieval simulation of a spatially uniform beam with an orbital angular momentum of 1. Fig. S10: effects of odd order aberration on the interferogram produced by the CDSI.

Supplementary 2 Video 1: this video shows the phase-shifting of the CDSI interferogram at a phase increment of 22.5 degrees.

Supplementary 3 Video 2: this video shows the typical shearing interferogram made up of an accumulation of 20, 40, 60, 80, 100, 200, 500, 1000, 5000, and 15000 frames.

Supplementary 4 Video 3: this video shows the comparison between Michelson interferometer and CDSI under vibration effects induced by a speaker.

Supplementary 5 Video 4: this video shows the polarization phase-shifting method by inserting a QWP before the CDSI and a polarizer after the CDSI. The polarizer is rotated at an increment of 11.25 degrees.

Supplementary 6 Video 5: this video shows the polarization effect on the visibility of the CDSI. The input polarization is a rotating linear polarization caused by rotating a half-wave plate at an increment of 11.25 degrees.
