# Supplementary material for: Spatial Wavefunction Characterization of Femtosecond Pulses at Single-Photon Level
Source: Research (Wash D C). 2020 Jun 15;2020:2421017. doi: 10.34133/2020/2421017 (PMC7312785; doi:10.34133/2020/2421017)
Supplement: Supplementary 1 — Table S1: measured probability p of all possible outcomes weighted on the probability of occurrence for photon number distribution. Fig. S4: phase shifting methods of the CDSI. Fig. S5: fringe pattern of the CDSI for a collimated beam recorded by the CCD camera when the image plane of the CCD lens and the entrance plane of the BSC are not conjugate of each other. Fig. S6: spatial wavefunction characterization of femtosecond-pulsed single photons with a sinusoidal wavefront shape. Fig. S7: spatial wavefunction characterization of CW single photons with a sinusoidal wavefront shape. Fig. S8: spatial wavefunction characterization of CW single photons with a triangular wavefront shape. Fig. S9: wavefront retrieval simulation of a spatially uniform beam with an orbital angular momentum of 1. Fig. S10: effects of odd order aberration on the interferogram produced by the CDSI. [file 2421017.f1.pdf]

Spatial wavefunction characterization of femtosecond pulses at single-photon level: Supplementary Materials

Billy Lam[1], Mohamed ElKabbash[1], Jihua Zhang[1], Chunlei Guo[1*]

[1]The Institute of Optics, University of Rochester, Rochester, New York 14627,USA

*E-mail: guo@optics.rochester.edu

May 26, 2020

MATERIALS AND METHODS

Experimental Design

The experimental setup is shown in Fig. 1 of the main manuscript. The details of the components in the setup as well as their settings during the experiment are described in the subsections titled 'Photon source', 'WRSI Assembly', and 'Imaging with ICCD camera.' The attenuated beam from the photon source propagates through the wedged-reversal shearing interferometer (WRSI) that consists of a single 50:50 beam splitter cube (BSC) with a wedged entrance face. The BSC is mounted on a 6-axis kinematic mount from Thorlabs controlling the shearing amount s_x and s_y by translation and the shearing direction by rotation, The mount also allows fine adjustments of the position and the tilt of the rotation axis. Prior to experiment, the BSC is aligned such that the hypotenuse face of the (BSC) is on the rotation axis and the rotation axis coincides with the optical axis of the beam. Please see Ref. 28 for the full procedure in aligning the CDSI. The output beams consisting of two interferograms are imaged onto an

intensified charge-coupled device (ICCD) camera (model iStar DH734). The gated pulse that activates the intensifier tube of the ICCD camera is set to the period of the ultrashort pulses. The probability of two photons landing on the same pixel is negligible. The acquired interferograms are accumulations of tens of thousands of frames. The interferograms with shearing amount of $s_x = 0, s_x = 300\mu m, s_y = 0$, and $s_y = 300\mu m$ at phase-shift of $0, \pi/2, \pi$ and $3\pi/2$ are obtained by rotating and translating the CDSI. The phases of these interferograms are manipulated numerically to extract the wavefront shape of the incident laser beam, which is at a single photon level unless stated otherwise. For a subset of the full wavefront information, in particular the odd order of x of the wavefront, only a subset of the mentioned interferograms are needed. After the data acquisition, they are processed using Matlab, a numerical computing software. The details of the formulas and the calculations involved for the wavefront extraction are provided in the 'Wavefront extraction' subsection.

Photon source

The photon source is a mode-locked oscillator of the Model MTS Mini Ti-Sapphire Laser Kit that generates ultrashort pulses with an 800 nm central wavelength, a 56 nm bandwidth, and 106 fs pulse duration at a 89 MHz repetition rate. The beam is attenuated by 9 orders of magnitude with ND-filters to the photon rate of $\sim$1 photon per pulse.

Imaging with ICCD camera

The ICCD camera used is of the model iStar DH734. Even though the minimum exposure time of the camera is 1 ms, the time resolution of this ICCD camera is determined by the 25 ps time resolution of the gated pulse that controls the intensifier tube. The gated pulse is set to be the pulse to pulse separation of the laser, which is 11.225 ps $\approx$ 1/89 MHz. The laser is attenuated such that the expected photon number in a pulse is approximately 1. The ICCD is

thermoelectrically cooled for noise reduction. Single photons can be resolved by increasing the gain sufficiently high to surpass the CCD readout noise while setting a threshold to be above the CCD readout noise in the photon counting mode. The image readout mode is used with a sub-region selected just large enough to capture the output beams. The vertical and horizontal binnings are set to be 8 to generate 'superpixel' array that sums data on-chip prior to readout and significantly reduce the chance for a single photon event to register in more than one pixel. This, in combination with sub-region-selection, increases the frame rate of the ICCD detector. The data are acquired in accumulation mode with the mentioned settings. A video showing the typical shearing interferogram made up of accumulation of 20, 40, 60, 80, 100, 200, 500, 1000, 5000, and 15000 frames is found in Visualization 2. The camera frame rate in this experiment is about 20 frames per second, so each set of measurement with 15000 frames takes about 750 seconds. The measurement time required for our method scales with the dimension d of the state, or the number of pixels.

Probability of undercounting

Undercounting occurs when two photons land on the same pixel in a single camera frame. This can be estimated using conditional probability of Prob($\geq$ 2 photons in 1 frame) $\times$ Prob(2 photons lands on same pixel|2 photons in 1 frame). The pulse energy of the oscillator is attenuated to 0.72 photons per camera frame for ultrashort pulses and 1.04 photons per camera frame for the CW beam. For a source that follows the Poisson distribution at such intensities, the probability of having 2 or more photons in a single frame is $1 -$ Prob(0 photon) $-$ Prob(1 photon) $= 0.1628$ for ultrashort pulses and 0.2790 for the CW beam. The gate pulse of the ICCD camera is set to the pulse-to-pulse separation of the oscillator. Therefore, each frame captures exactly one pulse. We now need to estimate the probability of two photons landing on the same pixel. According to the probability distribution of the beam profile measured in Fig. 2

of the ultrashort pulse, the pixel with the highest probability has a probability of 0.004, which is an overestimation of two photons landing on the same pixel given two photons in 1 camera frame. Similarly, this value is approximately 0.002 for the CW beam as shown in Fig. S7. Therefore, the probability of having two or more photons in a camera frame and two photons landing on the same pixel must be less than $0.1628 * 0.004 = 0.00065$ for ultrashort pulses and less than$0.2790 * 0.002 = 0.00056$ for the CW beam.

Operation principle of CDSI

As shown in figure 1 in the main manuscript, the final wavefunction at exit face 3 is the superposition of the left reflected portion and the right transmitted portion of the initial wavefunction. The optical path difference between the two portions can be written as

$$\phi(x, y) = (n - n_{air})t(y) + W(x, y) - W(-x - s, y) \tag{S1}$$

where n and n_{air} are the refractive index of the BSC and air respectively, $t(y) = 2\pi y \sin\alpha/\lambda + t(0) \approx 2\pi y\alpha/\lambda + t(0)$ is the thickness difference measured in radians of the two entrance faces resulted from the y-wedge angle α of face 2, and s is the shearing amount. The function $W(x, y)$ is the wavefront of the incident beam, which can be separated into odd and even order terms and then the terms containing the wavefront become $W_e(x, y) - W_e(x + s, y) + W_o(x, y) + W_o(x + s, y)$. To simplify the terms, we taylor expand the sheared wavefront as follows:

$$W(x + s, y) = W(x, y) + s\frac{\partial W(x, y)}{\partial x} + \frac{s^2}{2!}\frac{\partial^2 W(x, y)}{\partial x^2} + \mathcal{O}(s^3). \tag{S2}$$

The shearing amount s is chosen to be small for wavefront retrieval, so we keep only the two highest order terms of s as such:

$$W(x, y) - W(-x - s, y) = 2W_o(x, y) + s\frac{\partial W_o}{\partial x} - s\frac{\partial W_e}{\partial x}. \tag{S3}$$

Plugging this back into the optical path difference Eq. (S1) yields

$$\phi(x,y) = (n - n_{air})t(y) + s\frac{\partial W_o}{\partial x} - s\frac{\partial W_e}{\partial x} + 2W_o(x,y). \tag{S4}$$

Phase-shifting of the CDSI

The phase-shift of the interferogram (see Visualization 1) can be induced by a translation of the BSC along the wedge direction according to the first term in Eq. (2). With an effective wedge angle of 3.4 arcmin, a $y = 0.8$ mm translation of the BSC induces a π phase shift for the laser beam with a central wavelength of 800 nm (*28*). The second method is based on polarization phase-shifting, which requires a quarter-wave plate (QWP) and a polarizing BS. A QWP is inserted before the CDSI to change the incident polarization into circular polarization. The output beam is a vector sum of two collinear beams that are circularly polarized in the opposite sense. As a result, it possesses a polarization distribution that is entirely linear polarization whose direction depends on the optical path difference. Placing a polarizing BS after the CDSI will serve as a phase-shifter as the rotation θ of the linear polarizer induce a phase difference of 2θ between the transmitted linearly polarized components. Both methods are verified and the related results are shown in Fig. S4. For the polarization phase-shifting method, the fringes in both interferograms move in the opposite direction and the total energy out of a single output port of the polarizer is not conserved. This is why a polarizing BS must be used and both outputs must be measured for the accuracy enhancement. Both phase-shifting methods can be visualized in Visualization 1 and 4.

Michelson vs. CDSI on vibration effects

As mentioned previously, in comparison to the Michelson interferometer, our CDSI with this BSC is 3 orders of magnitude less vulnerable to vibration in the direction that causes phase shift.

Visualization 3 shows the direct comparison of the vibration effects on Michelson interferometer vs. CDSI. Both interferometers were mounted on speakers that run in series with a function generator as a source. The interferogram of the Michelson interferometer immediately vanishes when vibration is introduced while that of the CDSI had no visible difference. Vibration on the orthogonal directions has no effect for Michelson interferometers. For CDSI, vibration in the beam propagation direction has hardly any effect on the interferogram. Vibration in the shearing direction will change the shearing amount s and affect the interferogram that depends on the wavefront shape according to Eq. (2) in the main manuscript.

Avoiding unwanted diffraction effect

A very sharp beam cut off can be observed in the interferogram as in Fig. 2(a) - 2(h) of the main manuscript if the entrance plane of the BSC is imaged. Otherwise, diffraction will become pronounce and stripes parallel to the cut off can be seen. In addition, the beam cut off will smear out into tilted fringes as well. These diffraction effects are shown in Fig. S5. The intensity of the beam is adjusted beyond the saturation of the camera to better illustrate the diffraction effects. The CCD camera used to take this diffraction measurement is of the model Firefly MV FMVU-03MTM.

Wavefront extraction

The normalized 4-bin phase-shifting phase retrieval is based on four normalized interferograms described by:

$$Pr(x, y; 0) = I'(x, y) + I''(x, y) \cos(\phi(x, y)) \tag{S5}$$

$$Pr(x, y; \pi/2) = I'(x, y) - I''(x, y) \sin(\phi(x, y)) \tag{S6}$$

$$Pr(x, y; \pi) = I'(x, y) - I''(x, y) \cos(\phi(x, y)) \tag{S7}$$

$$Pr(x, y; 3\pi/2) = I'(x, y) + I''(x, y) \sin(\phi(x, y)) \tag{S8}$$

where $I'(x, y) = (|\Psi_i(-x - s, y)|^2 + |\Psi_i(x, y)|^2)/2$ is the average of the probability density of the two portions of the incident wavefunction on the CDSI, $I''(x, y) = |\Psi_i(-x - s, y)||\Psi_i(x, y)|$ is the geometric mean of the probability density of the two portions of the incident wavefunction, and $\phi(x, y)$ is the phase difference between the interfering beam. Each interferogram is normalized by dividing the total photon number, which is measured by capturing all outputs of an interferometer. Solving for the phase $\phi(x, y)$ yields

$$\phi(x, y) = \tan^{-1}\left(\frac{Pr(x, y; 3\pi/2) - Pr(x, y; \pi/2)}{Pr(x, y; 0) - Pr(x, y; \pi)}\right) \tag{S9}$$

which is Eq. (3) in the main manuscript.

After the phase retrieval, it's important to extract the wavefront. However, one should avoid using the technique of curve fitting in this step and manipulate the phase instead. The actual wavefront is maintained this way and further analysis using curving fitting of Zernike polynomials is available. For a typical shearing interferometer, wavefront extraction can easily be done by numerical integrating. The CDSI, however, requires a more sophisticated manipulation of the phase to extract the wavefront. The full procedure is as follows.

Recalls that the phase of the CDSI with the shearing direction of $\hat{\mathbf{x}}$ is

$$\phi^x(x, y; s) = c_0 + c_1 y + s\frac{\partial W_{ox}}{\partial x} - s\frac{\partial W_{ex}}{\partial x} + 2W_{ox}(x, y) \tag{S10}$$

where s is the shearing amount in the $+\hat{\mathbf{x}}$ direction, the subscript ox denotes odd order of x and ex denotes even order of x. The first two terms are due to the geometry of the setup where one

of the entrance faces of the beam splitter cube (BSC) has a y-wedge angle. By measuring the phase of an interferogram with $s = 0$, we will obtain

$$\phi^x(x, y; s = 0) = c_0 + c_1 y + 2W_{ox}(x, y). \tag{S11}$$

By Eq. (S11) and the property of odd function ($W_{ox}(0, y) = 0$), we can solve for the odd component of the wavefront. The solution is

$$W_{ox}(x, y) = \frac{\phi^x(x, y; s = 0) - \phi^x(0, y; s = 0)}{2} \tag{S12}$$

Now we isolate the derivative of the wavefront by subtracting Eq. (S10) by (S11):

$$\phi^x(x, y; s = s_x) - \phi^x(x, y; s = 0) = s_x \frac{\partial W_{ox}}{\partial x} - s_x \frac{\partial W_{ex}}{\partial x} \tag{S13}$$

Numerically integrating the above equation and dividing by s_x yields

$$W_{ox}(x, y) - W_{ex}(x, y) = \int_0^x \left(\frac{\phi^x(x', y; s = s_x) - \phi^x(x', y; s = 0)}{s_x} \right) dx' \tag{S14}$$

The even component of the wavefront can be computed by subtracting Eq. (S12) by Eq. (S14):

$$\begin{aligned} W_{ex}(x, y) = &\frac{\phi^x(x, y; s = 0) - \phi^x(0, y; s = 0)}{2} \\ &- \int_0^x \left(\frac{\phi^x(x', y; s = s_x) - \phi^x(x', y; s = 0)}{s_x} \right) dx' \end{aligned} \tag{S15}$$

The total wavefront is

$$W(x, y) = W_{ox}(x, y) + W_{ex}(x, y) + f(y) \tag{S16}$$

where $f(y) = W_{oy}(0, y) + W_{ey}(0, y)$. These wavefront components can be calculated using the interferogram with the shearing direction of $\hat{\mathbf{y}}$ following the same derivation above. Their expressions are

$$W_{oy}(0, y) = \frac{\phi^y(0, y; s = 0) - \phi^y(0, 0; s = 0)}{2} \tag{S17}$$

$$W_{ey}(0,y) = \frac{\phi^y(0,y;s=0) - \phi^y(0,0;s=0)}{2} - \int_0^y \left(\frac{\phi^y(0,y';s=s_y) - \phi^y(0,y';s=0)}{s_y} \right) dy' \quad \text{(S18)}$$

where s_y is the shearing amount in the $\hat{\mathbf{y}}$ direction and should be equal to s_x. Adding up all of the odd and even components in Eq. (S12, S15, S17, S18) will give us the total wavefront by Eq. (S16). However, because the interferograms of our CDSI only consists of half of the beam, we only obtain the total wavefront in the first quadrant. To obtain the wavefront in all four quadrants, we utilize the property of odd and even function to get

$$W(\pm x, y) = \pm W_{ox}(x,y) + W_{ex}(x,y) + W_{oy}(0,y) + W_{ey}(0,y) \quad \text{(S19)}$$

$$W(\pm x, -y) = \pm W_{ox}(x,y) + W_{ex}(x,y) - W_{oy}(0,y) + W_{ey}(0,y) \quad \text{(S20)}$$

where $x > 0$ and $y > 0$. As a summary, the wavefront in the four quadrants are extracted using the four phase distributions $\phi^x(x,y;s=0), \phi^x(x,y;s=s_x), \phi^y(x,y;s=0), \phi^y(x,y;s=s_y)$. If only the odd order wavefront are of interest, not all four phase distributions are needed. In particular, extracting $W_{ox}(x,y)$ ($W_{oy}(x,y)$) only requires one phase distribution $\phi^x(x,y,s=0)$ ($\phi^y(x,y,s=0)$).

The phase retrieval and wavefront extraction are done using our Matlab code that is available upon request. The Matlab code for phase unwrapping is taken from Ref. 38 and Ref. 39.

Elimination of noise from intensity fluctuation by normalization

In the main manuscript, we discussed how measuring over all the outputs in an interferometer and normalizing the measurements enhance the accuracy of measurement by eliminating the uncertainty due to photon number fluctuation. Here, we will show that the normalization results

in a phase sensitivity that scales as $N^{-1/2}$ independent of photon number fluctuation. Let $N \sim Pr(N = n)$ be the photon number distribution incident on the interferometer for a particular set of measurement conditions (integration time, quantum efficiency, incident beam) and $X \sim B(n, p_{i,j})/n$ be the normalized binomial distribution with n trials and $p_{i,j}$ being the probability that the photon lands on a particular pixel (x_i, y_j). From now on, the subscript i, j will be dropped. The measured probability p will be a compound binomial distribution:

$$Z = \sum_{n=1}^{\infty} Pr(N = n) \frac{B(n, p)}{n} \tag{S21}$$

Here we do not consider $n = 0$ as a measurement. The variance of the compound distribution is given by

$$Var(Z) = E(Var(X|N = n)) + Var(E(X|N = n)) \tag{S22}$$

Because the binomial distribution is normalized, the expected value of $X|N = n$ is equal to p for all n so the second term vanishes. The variance of Z simply becomes the weighted average of the variance of the normalized binomial distribution:

$$Var(Z) = \sum_{n=1}^{\infty} Pr(N = n) \frac{p(1 - p)}{n} \tag{S23}$$

Notice that for a particular photon number n, the variance is simply $Var(Z|N = n) = p(1 - p)/n$. Having known the photon number $N = n$ used in the experiment, the error in the measured value of p is $\sqrt{p(1 - p)/n}$. The accuracy of the measurement depends on the total number of photons used. A larger photon number yields a more accurate measurement of p: $[Var(Z|N = n_1) > Var(Z|N = n_2)]|(n_1 > n_2)$. It is important to note that the error $(\sqrt{Var(Z)})$ of the distribution Z is not to be confused with the error in the measured value of p when the number of photons is known after the measurement $(\sqrt{Var(Z|N = n)})$.

The measured probability p of each pixel achieves the best uncertainty one can achieve for whatever the number of photons used for the measurement regardless of the photon num-

ber fluctuation. To visualize this, consider the photon number distribution of $Pr(N = 1) = 0.5; Pr(N = 3) = 0.5$ and the probability of landing on the pixel is $p = 1/2$. Here we list out all the outcome of the experiment weighted on the probability of occurrence:

$N = 1$	$N = 3$
0/1	0/3
0/1	1/3
0/1	1/3
0/1	1/3
1/1	2/3
1/1	2/3
1/1	2/3
1/1	1/3

Table S1: Measured probability p of all possible outcome weighted on the probability of occurrence for photon number distribution of $Pr(N = 1) = Pr(N = 3) = 1/2$ and probability of a photon landing on the pixel $p = 1/2$

In this case, the error of the compound distribution $\sqrt{Var(Z)} = \sqrt{1/6}$ is the standard deviation of all of the sixteen numbers displayed in table S1. The error of the compound distribution does depend on the photon number fluctuation. However, if a particular measurement results in a photon number of 3, that measurement must belong to the second column ($B(3, 1/2)$) a binomial distribution with a total number of trials equal to 3 and a probability of success of $p = 1/2$. Hence, the error in the measured value of p is the standard deviation of the second column ($B(3, 1/2)$) and it equals $\sqrt{1/12}$. The error of the column of $N = 3$ is independent of the photon number fluctuation. Given that n photons actually registered during one such experiment, the error in the measured value of p is $\sqrt{p(1-p)/n}$, which is the same for light sources with different photon number statistics, whether it is Sub-Poissonian (*35*) or a delta function as in Fock state (*18*).

Accuracy enhancement by normalization for other types of interferometer

In general, this type of accuracy enhancement by normalization mentioned in the last section can be applied to most interferometric phase retrieval methods.In practice, certain interferometers require some minor modification in order to apply this accuracy enhancement. The setups must be modified to measure all the outputs of the interferometer. This is because the accuracy enhancement relies on the fact that the probability of the photons landing on the detector sums up to one. In particular, for polarization phase-shifting techniques that use polarizer (*36*), the polarizer must be replaced with a polarizing beam splitter and all of the outputs must be measured. For an interferometer with a back-propagating beam such as Michelson interferometer and Signac interferometer (*17*), a beam splitter (BS) must be placed before the interferometer to measure the back-propagating output. However, this also leads to an attenuation of the back-propagating beam so the other output must be attenuated by the same factor using an identical BS or simply by multiplying this factor in the result. This results in a probability distribution that adds up to a simple fraction that is independent of the phase distribution. For interferometers that utilize diffraction (*37*), the diffraction efficiency must be known. These spatially uniform losses are fundamentally identical to the effect of non-unitary quantum efficiency η at photodetectors. The pixelated probability distribution at the detector is still distributed identically, however, with values multiplied by η.

Additional data

Several videos are also supplied to aid the reader in visualizing how the interferogram evolves as certain parameters are changed. The Visualization 1 and 4 show the phase-shifting technique performed by translation of the BSC and the polarization phase-shifting technique respectively. The fringe visibility of the interferogram is affected by the incident polarization. The full detail is explained in Ref. 28. We also modulated this by inserting a half-wave plate (HWP) before the

CDSI. Visualization 5 illustrates this effect. This file shows the evolution of the fringe visibility as a function of HWP's fast axis at an increment of $11.25°$.

In addition to the triangular wavefront shape imprinted on the spatial light modulator (SLM) that is retrieved in Fig. 3 of the main manuscript, we have performed the same exact experiment with a sinusoidal wavefront shape. The related results are shown in Fig. S6. This experiment is also performed with a continuous wave (CW) laser beam at a single-photon level by breaking the mode-locking operation of the oscillator. The results are shown in Fig. S7. The experiment performed using a triangular wavefront shape and a CW laser beam at a single-photon level is shown in Fig. S8. The results of the experiments conducted using pulsed and CW operation are nearly identical except the beam size of the CW mode is slightly larger and the beam quality is not as Gaussian as the pulsed operation.

Simulations of complex spatial structures

A spatially uniform beam with an orbital angular momentum of 1 and its shearing interferogram with shear $s_x = 0$, $s_x = 1$ pixel, $s_y = 0$, and $s_y = 1$ pixel at four different phase-shifts of $0, \pi/2, \pi$, and $3\pi/2$, are simulated. The intensity profile is chosen to be uniform to make the fringes easy to recognize. The wavefront is then extracted using the method described in the section "wavefront extraction." The results are shown in Fig. S9. The retrieved wavefront is near identical to the simulated wavefront. However, the abrupt 2π phase change and the discontinuity at the singularity causes artifact along the $\hat{\mathbf{x}}$ direction. Excluding this artifact, the error is very close to zero.

CDSI as alignment tools

A major hurdle in any single-photon experiments lies in the alignment of the apparatus, which is very difficult and absolutely necessary. Because the CDSI can detect any asymmetric wavefront

at a shearing amount of $s = 0$, it can be used to verify whether optical elements are aligned perfectly along any single-photon beam including femtosecond-pulsed single photons. For example, the CDSI can be used to align lenses. Any change in propagation direction due to the insertion of a misaligned lens causes fringes to rotate because the wavefront tilt is the lowest order asymmetric wavefront aberration. A typical CDSI interferogram showing the tilt aberration is shown in Fig. S10 (a). If the beam is incident onto a lens with an oblique angle, it causes higher-order aberration that leads to curved fringes in the interferogram. All of these mentioned aberrations show up in the highest order term $2W_{ox}(x, y)$ in the optical path difference (Eq. (2)) of the main manuscript. The related results are shown in Fig. S10 (b-d) with different oblique angles. The full procedure of using the CDSI for aligning lenses is explained in Ref. 28.

The CDSI can align optical elements by minimizing not only the asymmetric wavefront of W_{ox} and W_{oy}, but also the symmetric wavefront of W_{ex} and W_{ey}. For example, the CDSI can check the collimation of the beam to check the alignment of 4f-systems. To detect any unwanted symmetric wavefront, the BSC can be translated along the shearing direction to change the shearing amount. The presence of any symmetric wavefront will cause the fringes to evolve during the BSC translation. The collimation testing has been demonstrated in Ref. 28.

Matlab data files and codes

All of the data in this paper are processed using Matlab. The Matlab data files and codes may be requested from the authors. The function unwrap_phase that is used in most of the codes is developed by Kasim, et al. (*38, 39*).

Statistical Analysis

The gated pulse of the intensifier tube that intensifies and collects optical signal is set to be the period of the ultrashort laser pulses. Thus, the attenuated pulses follow a Poisson distribution

with a photon rate of about η photons per frame where η is the quantum efficiency of the ICCD camera. The probability of landing on a particular pixel in the detector is described by the probability density in Eq. (1) of the main manuscript after normalization:

$$\begin{aligned} K|\Psi(x,y)|^2 = K(&|\psi_i(-x,y)|^2 + |\psi_i(x-s,y)|^2 \\ &- 2|\psi_i(-x,y)||\psi_i(x-s,y)|\cos(\phi(x,y))) \qquad x \geq d \end{aligned} \tag{S24}$$

where K is the normalization constant such that the probability sums up to 1 over all the pixels.

The error in measured value of p of each pixel is $\sqrt{p(1-p)/N}$ where N is the total photon number measured over both output of the BSC for each measurement. The phase is retrieved from four interferograms using:

$$\phi(x,y) = \tan^{-1}\left(\frac{Pr(x,y;3\pi/2) - Pr(x,y;\pi/2)}{Pr(x,y;0) - Pr(x,y;\pi)}\right) \tag{S25}$$

We use the variance formula of error propagation to find the absolute error as follows [29]:

$$\Delta\phi(x,y) = \sqrt{\sum_{i=0}^{3}\left(\frac{d\phi(x,y)}{dPr(x,y,i\pi/2)}\right)^2 [\Delta Pr(x,y,i\pi/2)]^2} \tag{S26}$$

The derivative can be easily calculated using chain rule, and the trigonometric identities $d\tan^{-1}(\theta)/d\theta = 1/(1+\theta^2)$. The absolute error was calculated in the main manuscript which is just the standard deviation of a normalized binomial distribution. The expression becomes

$$\Delta\phi = \sqrt{\sum_{i=0}^{3}\frac{Pr(i\pi/2)[1-Pr(i\pi/2)][1+\tan\phi+(-1)^i(1-\tan\phi)]^2}{4N_k(1+\tan^2\phi)^2(Pr(x,y;0)-Pr(x,y;\pi))^2}} \tag{S27}$$

where N_k is the total number of photons over all outputs used for the measurement of $Pr(x,y,i\pi/2)$. It is of great interest to determine the noise limit. In fact, the phase noise in Eq. (S27) scales as $1/\sqrt{N_k}$, the standard quantum limit.

Reaching the standard quantum limit

For any type of phase retrieval that involves multiple measurement of intensity (probability) distribution, normalization results in a phase error of [29]:

$$\Delta\phi(x,y) = \sqrt{\sum_{i=1}^{i_{max}} \left(\frac{d\phi(x,y)}{dPr_k(x,y)}\right)^2 [\Delta Pr_k(x,y)]^2} \tag{S28}$$

as derived in Eq. (5) of the main manuscript. The absolute error of $\Delta Pr_k(x,y)$ is $\sqrt{Pr_k(x,y)(1-Pr_k(x,y))/N_k}$ where N_k is the total number of photons over all outputs used for the measurement of $Pr_k(x,y)$. The derivative term is independent of N_k because both $\phi(x,y)$ and the probability distribution $Pr_k(x,y)$ are independent of N_k. Given that the total number of photons used of each intensity measurement are the same, the phase noise $d\phi(x,y)$ scales as $1/\sqrt{N_k}$, the standard quantum noise limit.

There is a special case in which the standard quantum noise limit is achieved. This is when the visibility is one and there is only one pixel on each of the detectors over two complement outputs of an interferometer. Because there is only a single pixel over an output, the phase needs to be spatially uniform. For such a configuration, the photon count over an output can be described by the equation $N_1 = N * p_1 = (N + N\cos\phi)/2$, where N_1, N is the total number of photons registered over detector 1, both detectors, respectively. The phase uncertainty can be calculated as follows:

$$\Delta\phi = \left|\frac{\Delta N_1}{\frac{dN_1}{d\phi}}\right| \tag{S29}$$

Among the series of N photons, the N_1 photons that landed on an output can be modeled by a binomial distribution. This allows us to calculate the right hand side of Eq. (S29), resulting in

$$\Delta\phi = \left|\frac{\sqrt{Np_1(1-p_1)}}{-\frac{N}{2}\sin\phi}\right| \tag{S30}$$

Substituting $p_1 = (1+\cos\phi)/2$, and $\sin\phi = \sqrt{1-\cos^2}$ yields exactly $\Delta\phi = 1/\sqrt{N}$.

Number-phase uncertainty relation

The number-phase uncertainty relation (*27*) states that

$$\Delta n \Delta \phi \geq \frac{1}{2} \tag{S31}$$

The amplitude and wavefront retrieval seems to violate this uncertainty relation. However, the shearing interferometer only measures the phase difference of the two beams in the transverse distribution. This phase difference contains the term $W(x,y) - W(-x-s,y)$ as shown in Eq. (S1). It does not measure the absolute phase because the phase is completely insensitive to the absolute phase. After retrieving the wavefront from the phase difference, we obtain the quantity $(W(x,y) - W(0,0))$ by integration that describes the shape of the wavefront while $W(0,0)$ is unknown. Thus, $W(x,y)$ is completely unknown. We simply set $W(0,0) = 0$ in the final result because the constant offset is not important. Therefore, it is possible to know the shape of the wavefront precisely even for Fock state while the absolute phase remains completely unknown.

References

35. J. Rarity, P. Tapster, E. Jakeman, ”Observation of sub-poissonian light in parametric down-conversion,” *Optics communications*, vol. 62, no. 3, pp. 201-206, 1987.

36. M. P. Kothiyal, and C. Delisle, ”Shearing interferometer for phase shifting interferometry with polarization phase shifter,” *Applied Optics*, vol. 24, no. 24, article 4439, 1985.

37. J. C. Wyant, ”Double frequency grating lateral shear interferometer,” *Applied optics*, vol. 12, no. 9, pp 2057-2060, 1973.

38. M. A. Herraez, D. R. Burton, M. J. Lalor, and M. A. Gdeisat, "Fast two-dimensional phase-unwrapping algorithm based on sorting by reliability following a noncontinuous path," *Applied Optics*, vol. 41, no. 35, pp. 7437-7444, 2002.

39. M. F. Kasim, "Fast 2D phase unwrapping implementation in matlab," 2017, https://github.com/mfkasim91/unwrap_phase/.

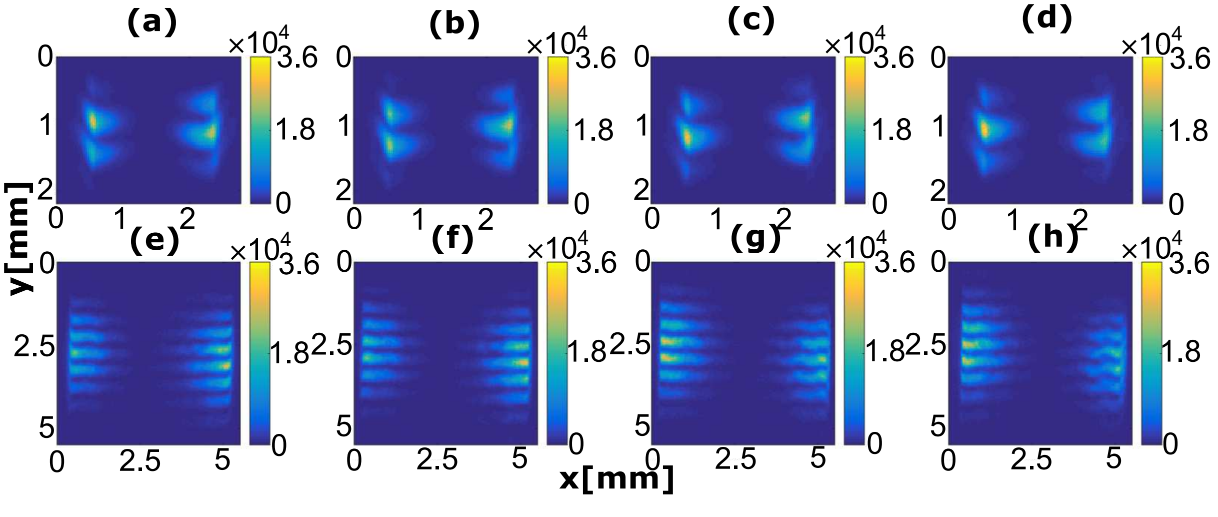

Figure S4: Phase shifting methods of the CDSI. (a-d) Interferograms provided by CDSI with shearing amount $s_x = 0$ using a collimated s-polarized beam after translating the BSC by (a) 0 mm (b) 0.20 mm (c) 0.40 mm (d) 0.60 mm along the x-axis. (e-f) Interferograms provided by CDSI with shearing amount $s_x = 0$ using a collimated circularly polarized beam with output polarizer at angle of (e) 112.5°, (e) 135°, (e) 157.5°, (e) 180°. The data are acquired using a long exposure of a few seconds before saturation happens.

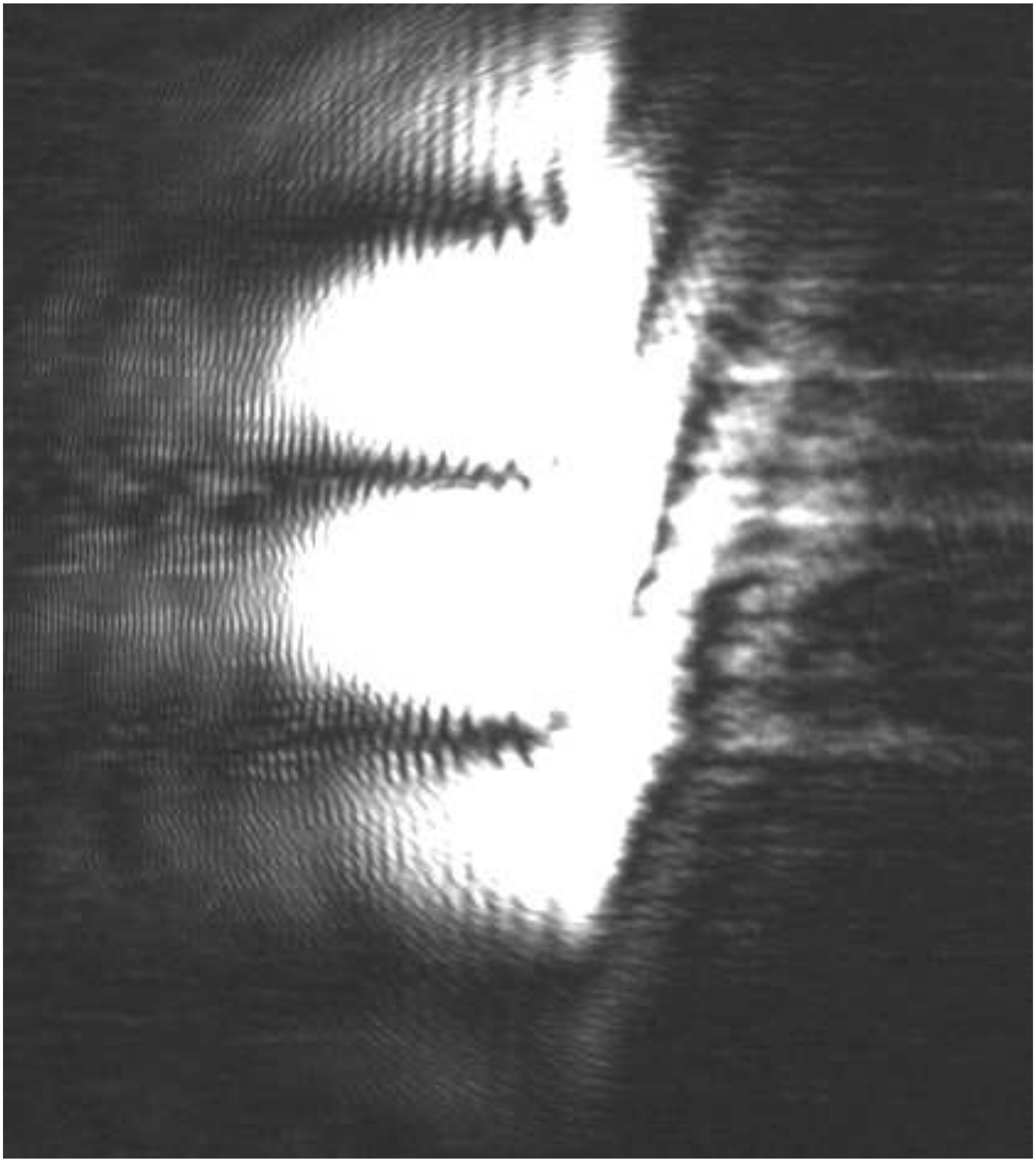

Figure S5: fringes pattern of the CDSI for a collimated beam recorded by the CCD camera when the image plane of the CCD lens and the entrance plane of the BSC are not conjugate of each other. The photon rate is increased to 1000 photons/meter to better show the diffraction effects.

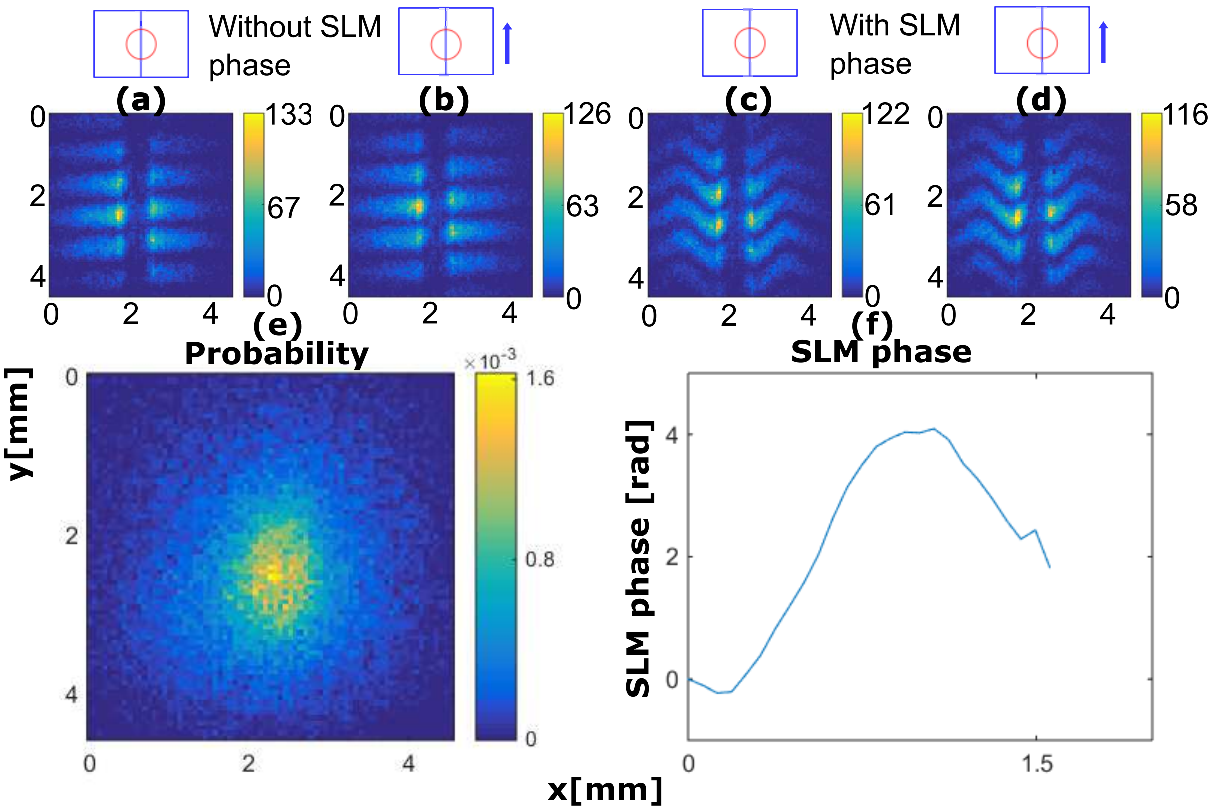

Figure S6: Spatial wavefunction characterization of femtosecond-pulsed single photons with a sinusoidal wavefront shape. The 4-bin phase shifting (induced by translation of BSC) is applied to the interferograms with a shearing amount of $s_x = 0$ to retrieval the phases. The phases are manipulated numerically to extract the one-dimensional odd-order SLM phase distribution. (a-d) Interferograms produced by a collimated beam going through the CDSI at $s_x = 0$ (a, b) without SLM phase modulation and (c, d) with SLM sinusoidal phase modulation. The phase shift pairs are (a, c) 0 and π, and (b, d) $\pi/2$ and $3\pi/2$. (e) The probability distribution of the single-photon measured by translating the BSC to fully contain the beam. (j) Extracted SLM phase based on the interferograms (a-d). Each interferogram is made up of an accumulation of 60000 frames at the rate of 0.72 photons per frame.

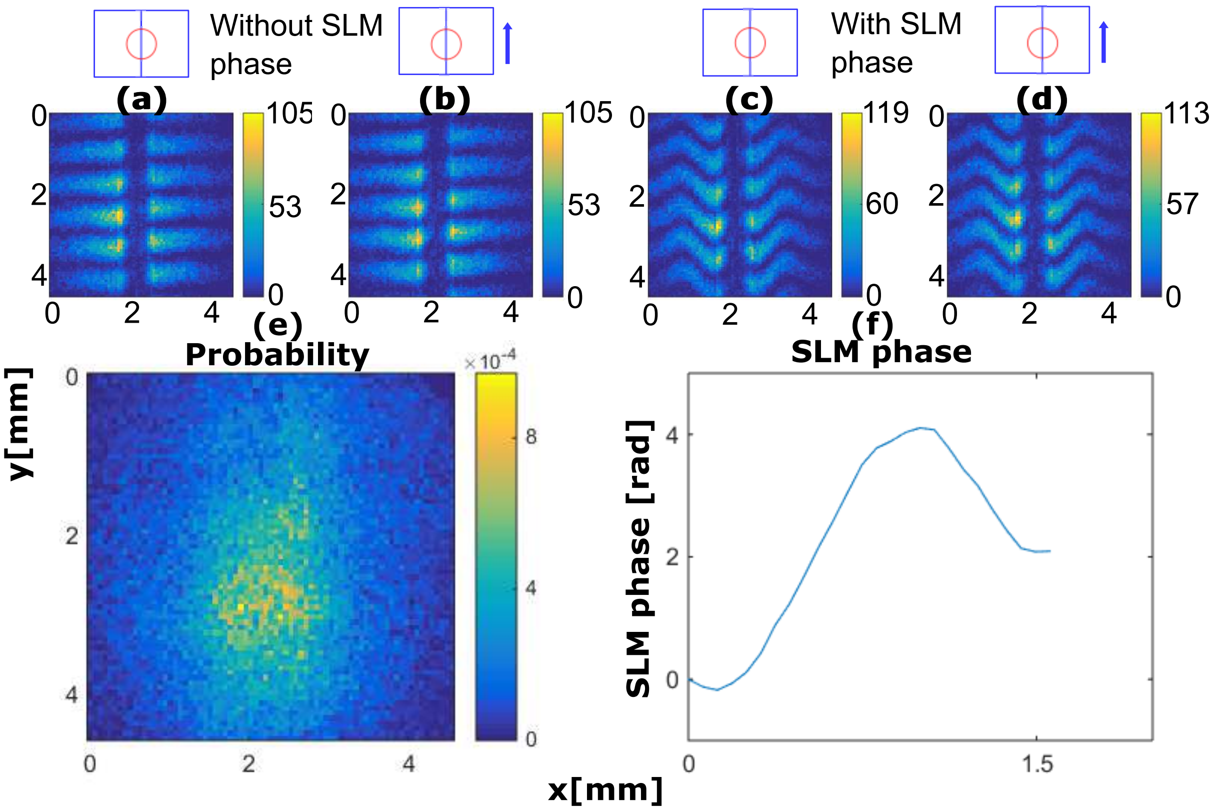

Figure S7: Spatial wavefunction characterization of CW single photons with a sinusoidal wavefront shape. The 4-bin phase shifting (induced by translation of BSC) is applied to the interferograms with a shearing amount of $s_x = 0$ to retrieval the phases. The phases are manipulated numerically to extract the one-dimensional odd-order SLM phase distribution. (a-d) Interferograms produced by a collimated beam going through the CDSI at $s_x = 0$ (a, b) without SLM phase modulation and (c, d) with SLM sinusoidal phase modulation. The phase shift pairs are (a, c) 0 and π, and (b, d) $\pi/2$ and $3\pi/2$. (e) The probability distribution of the single-photon measured by translating the BSC to fully contain the beam. (j) Extracted SLM phase based on the interferograms (a-d). Each interferogram is made up of an accumulation of 60000 frames at the rate of 1.04 photons per frame.

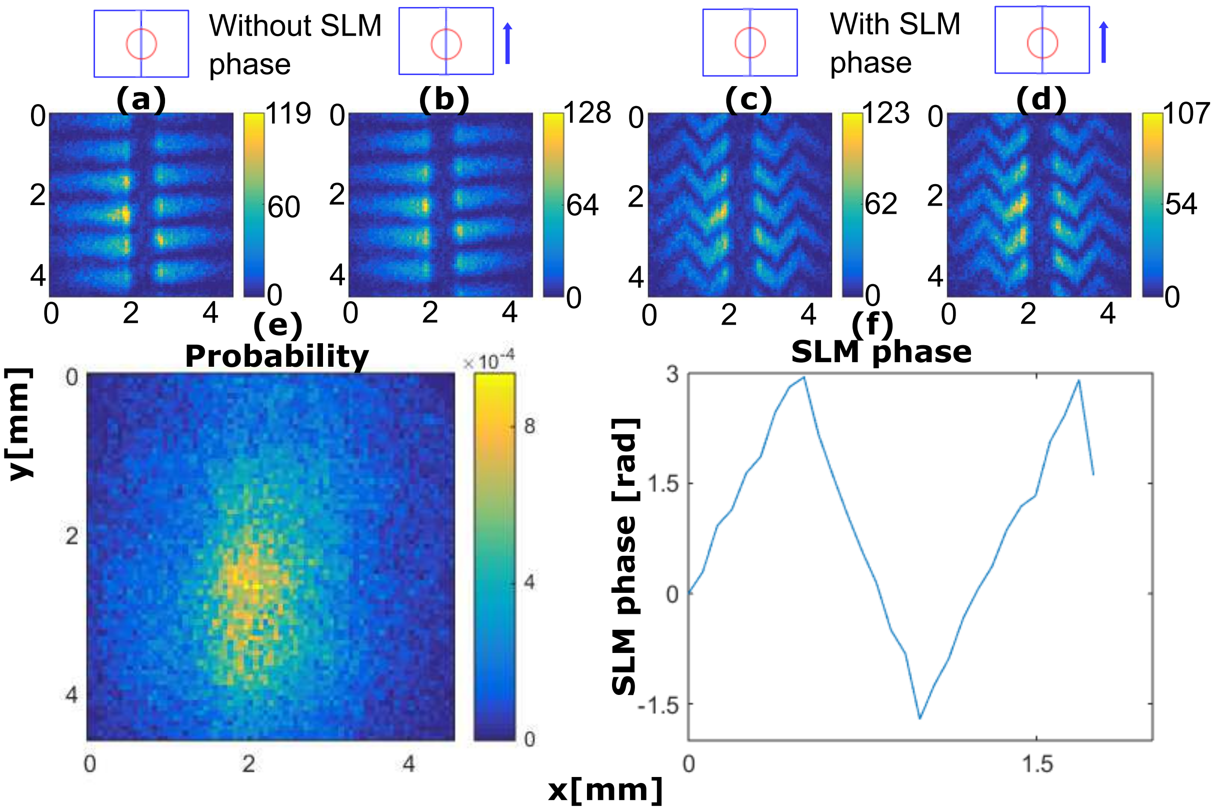

Figure S8: Spatial wavefunction characterization of CW single photons with a triangular wavefront shape. The 4-bin phase shifting (induced by translation of BSC) is applied to the interferograms with a shearing amount of $s_x = 0$ to retrieval the phases. The phases are manipulated numerically to extract the one-dimensional odd-order SLM phase distribution. (a-d) Interferograms produced by a collimated beam going through the CDSI at $s_x = 0$ (a, b) without SLM phase modulation and (c, d) with SLM triangular phase modulation. The phase shift pairs are (a, c) 0 and π, and (b, d) $\pi/2$ and $3\pi/2$. (e) The probability distribution of the single-photon measured by translating the BSC to fully contain the beam. (j) Extracted SLM phase based on the interferograms (a-d). Each interferogram is made up of an accumulation of 60000 frames at the rate of 1.04 photons per frame.

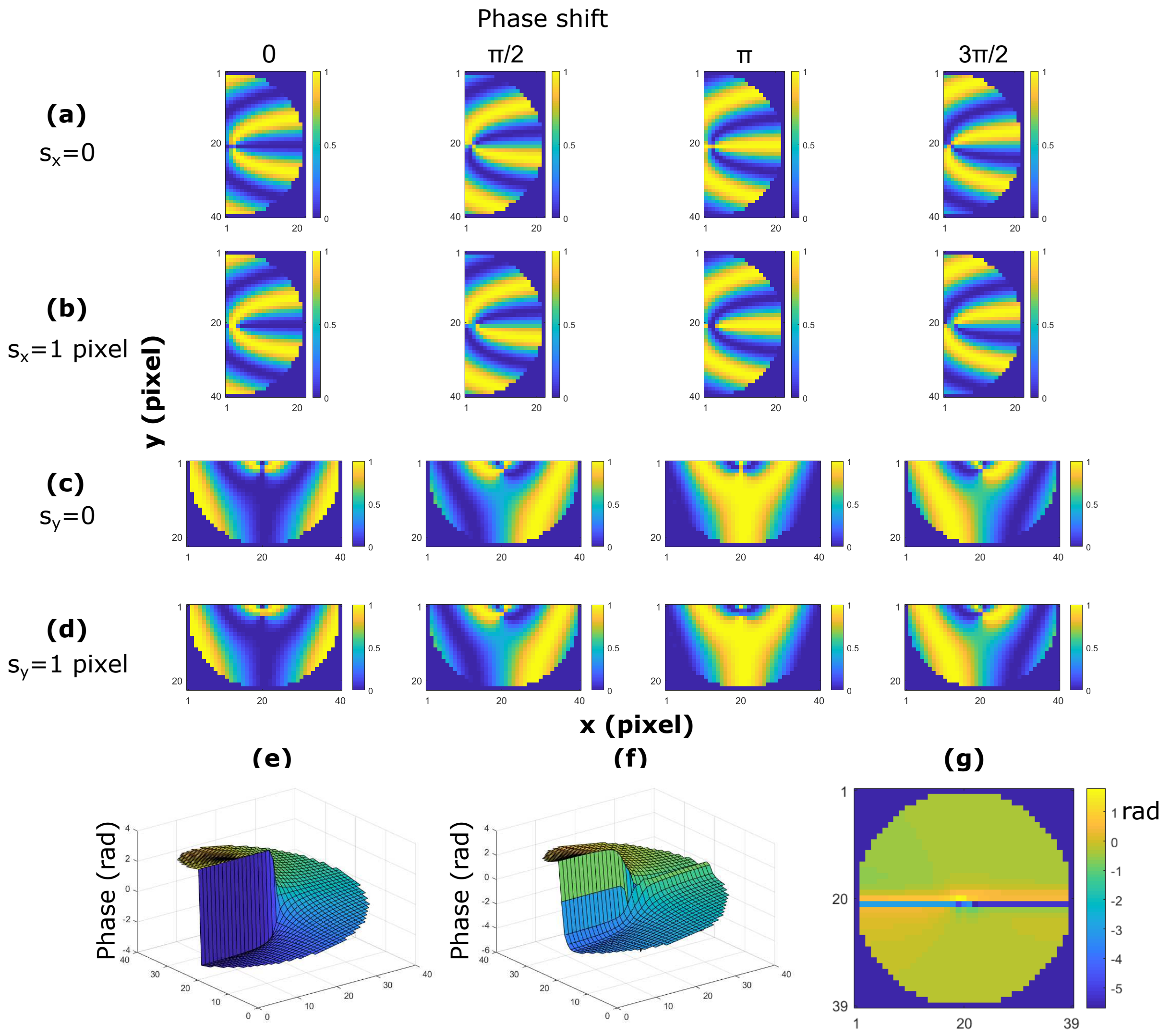

Figure S9: Wavefront retrieval simulation of a spatially uniform beam with an orbital angular momentum of 1. (a-d) Shearing interferogram with shear (a) $s_x = 0$, (b) $s_x = 1$ pixel, (c) $s_y = 0$, and (d) $s_y = 1$ pixel. Four different phase-shifts of $0, \pi/2, \pi$, and $3\pi/2$ are displayed from left to right. (e) Simulated wavefront with an orbital angular momentum of 1. (f) Extracted wavefront based on the interferograms (a-d). (g) error of the extracted wavefront.

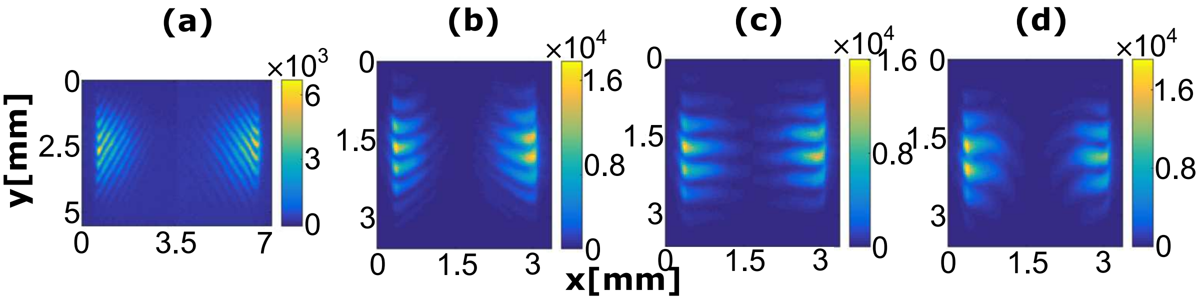

Figure S10: Effects of odd order aberration on the interferogram produced by the CDSI. (a) Interferogram produced by the CDSI using a beam with x-tilt. (b-d) Interferograms produced by CDSI with shearing amount $s_x = 0$ using a collimated beam that passed through a plano-convex lens (f = 250 mm) with oblique angle of (b) -26°, (c) 0°, (d) -26°. These data are not acquired in photon counting mode.
